# Molecular magnetic resonance imaging of brain–immune interactions

**DOI:** 10.3389/fncel.2014.00389

**Published:** 2014-11-27

**Authors:** Maxime Gauberti, Axel Montagne, Aurélien Quenault, Denis Vivien

**Affiliations:** Inserm, Inserm UMR-S U919, Serine Proteases and Pathophysiology of the Neurovascular Unit, Université de Caen Basse-Normandie – GIP CyceronCaen, France

**Keywords:** inflammation, stroke, Alzheimer, multiple sclerosis, hemorrhage, lymphocytes, microparticles, antibody

## Abstract

Although the blood–brain barrier (BBB) was thought to protect the brain from the effects of the immune system, immune cells can nevertheless migrate from the blood to the brain, either as a cause or as a consequence of central nervous system (CNS) diseases, thus contributing to their evolution and outcome. Accordingly, as the interface between the CNS and the peripheral immune system, the BBB is critical during neuroinflammatory processes. In particular, endothelial cells are involved in the brain response to systemic or local inflammatory stimuli by regulating the cellular movement between the circulation and the brain parenchyma. While neuropathological conditions differ in etiology and in the way in which the inflammatory response is mounted and resolved, cellular mechanisms of neuroinflammation are probably similar. Accordingly, neuroinflammation is a hallmark and a decisive player of many CNS diseases. Thus, molecular magnetic resonance imaging (MRI) of inflammatory processes is a central theme of research in several neurological disorders focusing on a set of molecules expressed by endothelial cells, such as adhesion molecules (VCAM-1, ICAM-1, P-selectin, E-selectin, …), which emerge as therapeutic targets and biomarkers for neurological diseases. In this review, we will present the most recent advances in the field of preclinical molecular MRI. Moreover, we will discuss the possible translation of molecular MRI to the clinical setting with a particular emphasis on myeloperoxidase imaging, autologous cell tracking, and targeted iron oxide particles (USPIO, MPIO).

## INTRODUCTION

Brain–immune interactions play a central role in acute neurological disorders including ischemic stroke, intracranial hemorrhage and traumatic brain injury (Wang and Dore, 2007; Iadecola and Anrather, 2011; Woodcock and Morganti-Kossmann, 2013). Given the proven and putative clinical benefits of modulating brain–immune interactions, non-invasive methods aimed at imaging the molecular players involved in these processes have been the subject of numerous studies. The most promising approaches to visualize brain–immune interactions are magnetic resonance imaging (MRI), positron emission tomography (PET), single photon emission computed tomography (SPECT), and optical imaging (in preclinical studies). Only molecular MRI combines fast acquisition time, wide clinical availability and acceptable safety profile for repeated scanning. However, molecular MRI lacks sensitivity compared to PET or SPECT. Notably, recent developments in the field of MRI contrast agent dramatically improved MRI sensitivity and allowed, for the first time, reliable imaging of the proteins involved in brain immune interactions with high spatial and temporal resolutions.

Brain–immune interactions occur all over the time course of acute neurological diseases. Upon injury, endothelial cells of the cerebrovasculature become activated and release the content of their Weibel-Palade bodies. This leads to P-selectin exposure on their surface and subsequent adhesion of neutrophils through P-selectin – P-selectin Glycoprotein Ligand-1 (PSGL-1) interactions (Ley et al., 2007). In parallel, in the brain parenchyma, an early phase of microglial activation is followed by a local inflammatory response (including microgliosis, astrogliosis, and cytokines/chemokines secretion) which sustains the activation state of brain endothelial cells. Expression of E-selectin, intercellular adhesion molecule-1 (ICAM-1), and vascular cell adhesion molecule-1 (VCAM-1) on the luminal endothelial surface allows adhesion and subsequent diapedesis of systemic immune cells inside the brain parenchyma. These infiltrated cells further enhance the inflammatory reaction in order to fight potential pathogens and clear cellular debris. To this aim, they secrete numerous cytokines/chemokines, proteolytic enzymes, and peroxydases (including myeloperoxidase, MPO). Neuroinflammation is also deemed necessary for the post-injury reparation phase that can last for weeks after the initial brain insult (Kyritsis et al., 2012; Tobin et al., 2014). This phase involves neurogenesis, dendritogenesis, oligodendrogenesis, axon sprouting, and matrix remodeling to restore tissue integrity (Peruzzotti-Jametti et al., 2014). All these mechanisms are common to acute brain injuries (including ischemia, hemorrhage, and trauma), neuroinflammatory (including multiple sclerosis) and neurodegenerative disorders (Schwartz et al., 2013). However, discrepancies exist in terms of kinetic, localization, and intensity of the inflammatory responses.

Improvement in our general knowledge of the brain–immune interactions taking place during acute neurological disorders revealed new potential therapeutic targets. Nevertheless, the successes and failures of immunomodulatory treatments for acute brain injury remind us that precise characterization of the interplay between leukocytes, endothelial adhesion molecules, glial, and neuronal cells is mandatory for efficient design of therapeutic strategies targeting brain–immune interactions. For instance, Natalizumab, a monoclonal antibody targeting the interaction between very late antigen 4 (VLA-4) and VCAM-1, has been successfully developed as a treatment for multiple sclerosis (Miller et al., 2003). In contrast, Enlimomab, a monoclonal antibody targeting neutrophils diapedesis by ICAM-1 blockade, has failed in phase III trials involving ischemic stroke patients (Enlimomab Acute Stroke Trial, 2001), despite preclinical evidence of its potent neuroprotective effects (Matsuo et al., 1994; Connolly et al., 1996). In fact, contrary to what initially thought, a recent study demonstrated that neutrophils do not reach the brain parenchyma after stroke in humans (Enzmann et al., 2013). Moreover, it becomes now clearer that one size does not fit all: variability in individual inflammatory response following acute brain injury suggests that modulation of brain–immune interaction may prove beneficial in some individuals but not in others. Considering that blockade of leukocytes subsets may also be associated with impaired host defense against pathogens, adequate selection of patients should be performed before administration of immunomodulatory drugs.

**FIGURE 1:**
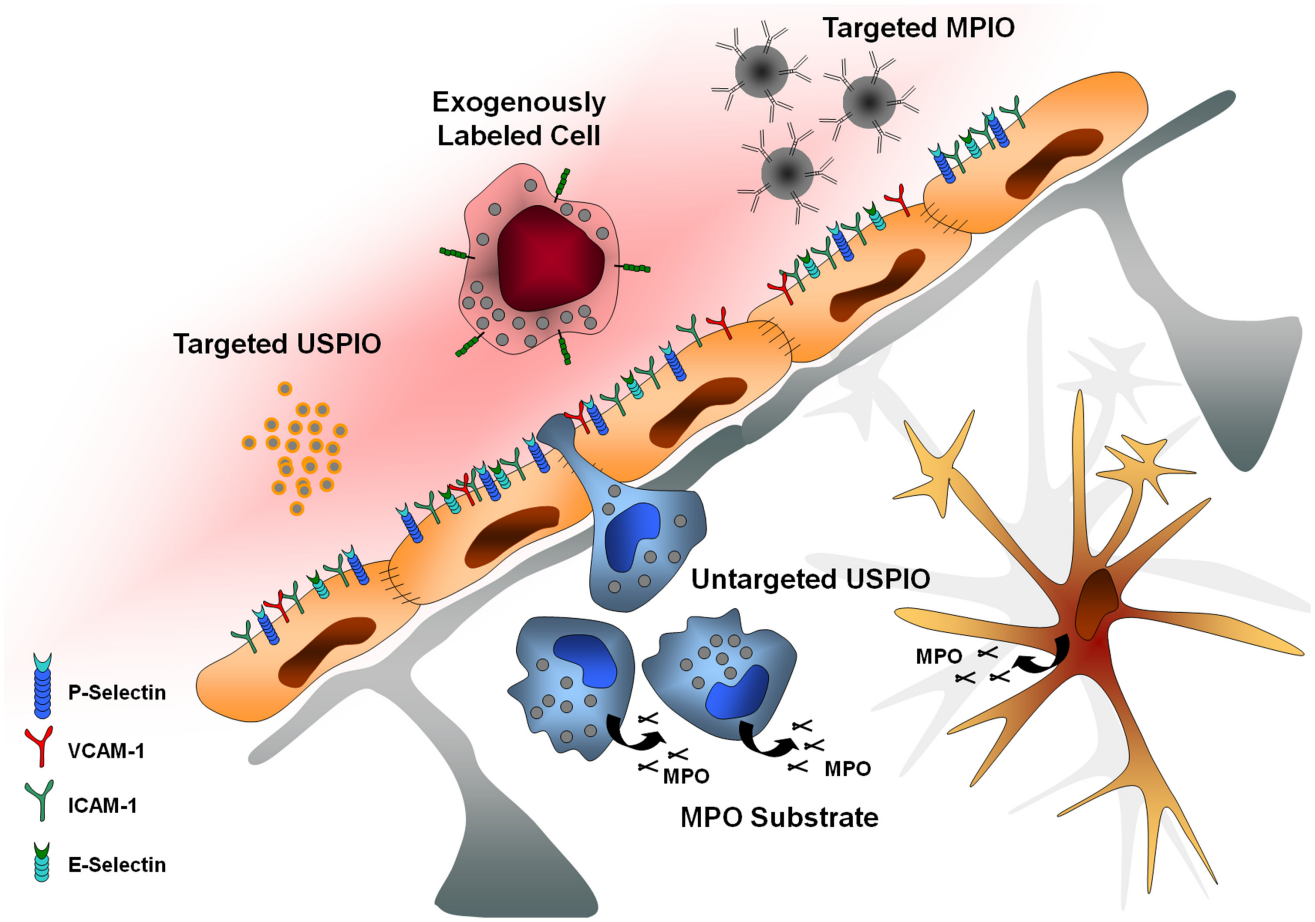
**Main targets of molecular imaging.** USPIO, ultrasmall particles of iron oxide; MPIO, microsized particles of iron oxide; MPO, myeloperoxidase; VCAM-1, vascular cell adhesion molecule-1; ICAM-1, intercellular adhesion molecule-1.

In this context, molecular MRI appears highly promising to select patient candidates for immunomodulatory treatments, monitor treatment efficiency, and more generally improve our knowledge on brain–immune interactions in human patients. The purpose of this review is to present recent advances in the field of molecular MRI of brain–immune interactions. After presentation of the theoretical bases of molecular MRI and of the brain-specific issues of this non-invasive method, we will present recent studies relevant to brain–immune interactions after acute cerebral injury with a particular focus on MPO imaging, autologous cell tracking, unlabeled ultrasmall superparamagnetic particles of iron oxide (USPIOs), targeted USPIOs, and targeted microsized particles of iron oxide (MPIOs; **Figure 1**).

## BASIS OF MOLECULAR MAGNETIC RESONANCE IMAGING

**FIGURE 2:**
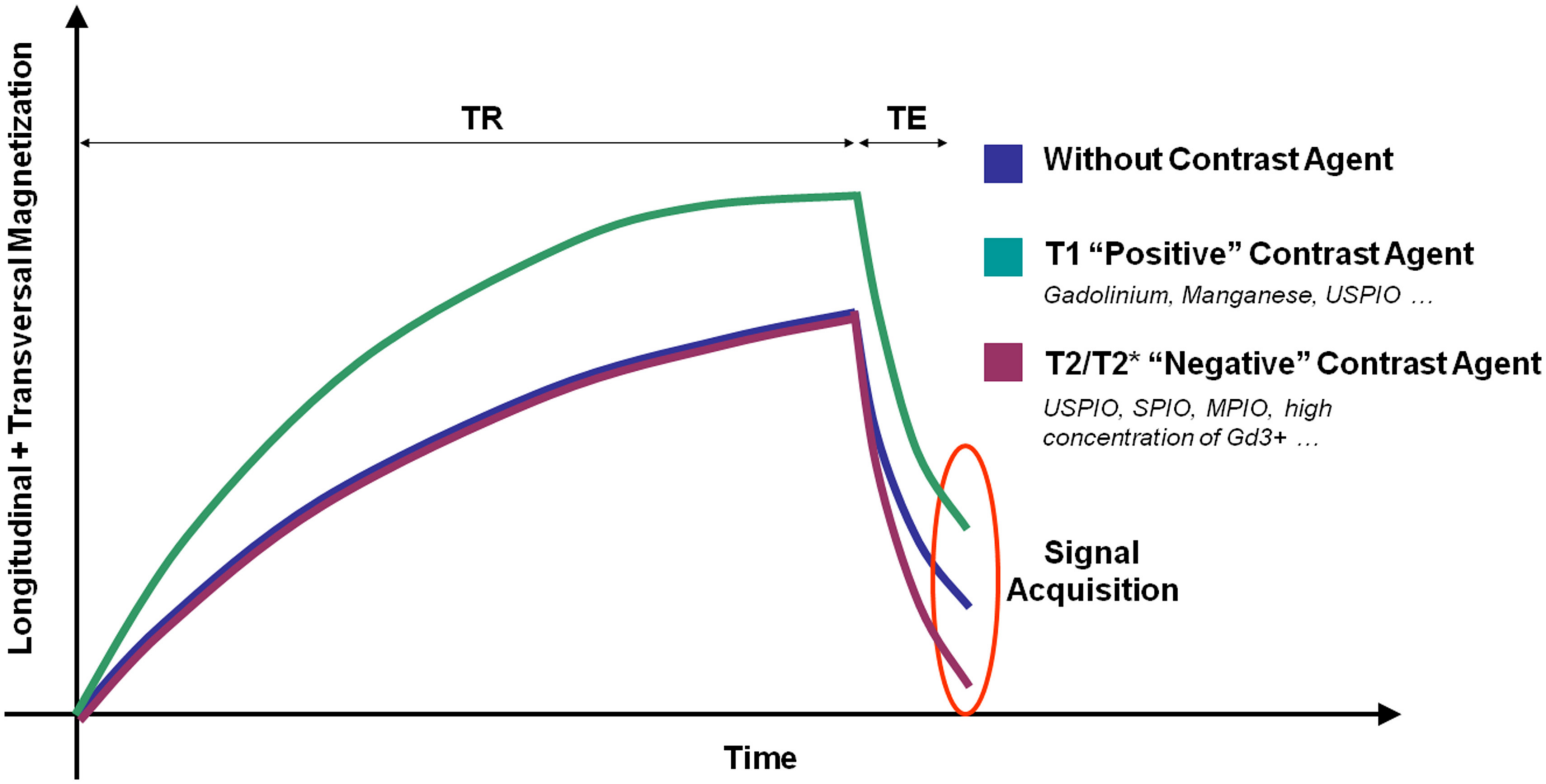
**Schematic representation of the effects of different contrast agents on water magnetization in biological samples.** TR, repetition time; TE, Echo Time.

Magnetic resonance imaging signal is generated by the magnetization of the hydrogen nuclei (protons) from biological tissues. Basically, two different classes of MRI contrast agents are available: T1 contrast agents increase the signal on T1-weighted images by shortening the spin-lattice relaxation time (T1 constant). T2 and T2* contrast agents decrease the signal on T2- and T2*-weighted images by both shortening the spin–spin relaxation time (T2 constant) and dephasing adjacent protons (by modification of their precession angular velocity). Therefore, depending on the contrast material injected, a molecular target can be revealed by an increase (paramagnetic T1 contrast agent, mainly Gadolinium-based) or a decrease (superparamagnetic T2 or T2* contrast agent, mainly iron-oxide-based) in the MRI signal (**Figure 2**). New methods of molecular MRI have been recently developed [such as chemical exchange saturation transfer (CEST), positive contrast imaging or MRI of non-hydrogen nuclei] but they still present a limited sensitivity compared to more conventional methods. Their development remains however of great interest since such contrast agents could allow imaging multiple targets simultaneously (Woods et al., 2006).

Independently of the contrast agent, the general concept of molecular MRI is to target a contrastophore (either Gadolinium- or Iron-oxide-based) to a protein of interest using a targeting moiety (pharmacophore). This targeting moiety can be either a specific ligand (such as a protein, an antibody or a specifically designed peptide) or a substrate of an enzyme which can accumulate in the regions where its corresponding enzyme is overexpressed/active. For instance, VCAM-1 imaging can be performed using microparticles of iron oxide (MPIO, the contrastophore) coupled to monoclonal antibodies targeting VCAM-1 (the pharmacophore). After intravenous injection, this agent binds to endothelial VCAM-1 and induces T2* effects leading to decreased signal on T2*-weighted images (i.e., signal voids) in regions presenting activated endothelial cells (McAteer et al., 2007).

**FIGURE 3:**
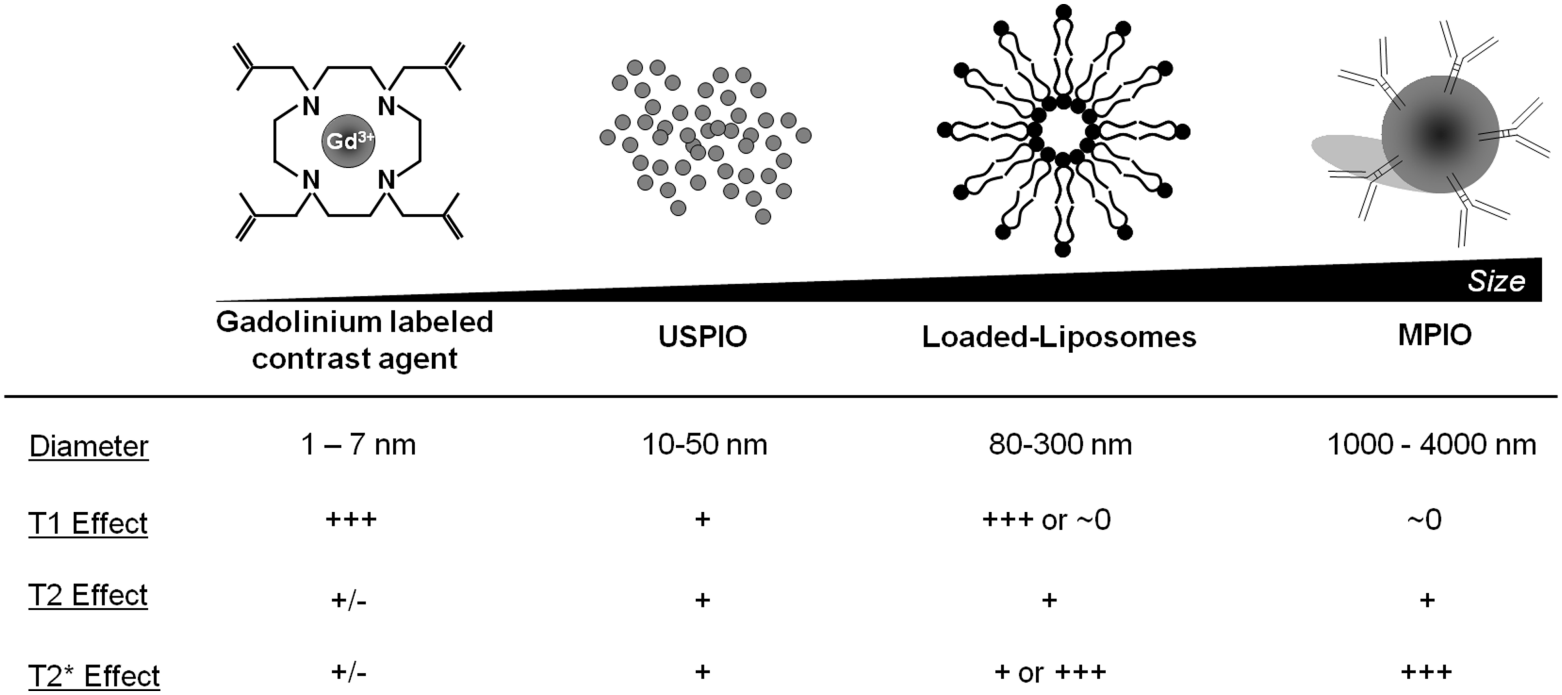
**Main characteristics of the most widely used contrastophores**.

The concentration of contrast material is one of the most important limits of molecular MRI. Indeed, whereas PET can detect β^+^ emitting atoms at picomolar concentration, MRI using classical Gadolinium-based contrast agent (GBCA) presents a sensitivity in the micromolar range. To overcome this limitation, contrast agent carrying large payload of gadolinium or iron oxide have been developed, including paramagnetic liposomes and iron oxide particles (**Figure 3**). In this regard, MPIOs (1–4 μm in diameter) are particularly interesting since even a single particle can be detected by high-field MRI at high resolution (Montagne et al., 2012). After binding to its target, targeted MPIOs induce dephasing of the surrounding protons, leading to strong and large T2* effects, up to 50 times larger than the original particle size (∼50 μm; Shapiro et al., 2006). At equal concentrations of iron, MPIOs induce much stronger T2* effects than USPIOs (10–50 nm; Montagne et al., 2012). This new generation of contrastophores is therefore especially efficient for the detection of targets present at low concentration on the surface of brain endothelial cells.

The use of high resolution and strongly T2*-weighted images to improve MRI sensitivity may also favor endogenous contrasts and false positive detection of iron oxide particles. In particular, hemorrhages and blood oxygen level dependent (BOLD) effects induce signal voids on T2*-weighted images which are comparable to MPIO-induced signal void and could therefore lead to false positive results. To overcome this, we demonstrated in a recent study in mice that a short preparation time (30 min) of normobaric hyperoxia can be sufficient to transform deoxyhemoglobin (paramagnetic) into oxyhemoglobin (diamagnetic) by increasing the tissular concentration of O_2_ (Gaberel et al., 2013). Thus, the susceptibility effect of endogenous blood is blunted, allowing observing changes in signal intensity that are specific to the contrast agent injected. Importantly, this preparation period of hyperoxia did not impair behavioral testing in mice presenting intracranial hemorrhages suggesting that this strategy is safe. Further studies should look at the effect of normobaric hyperoxia on T2*-weighted images in larger animals and humans.

## BRAIN SPECIFIC ISSUES IN MOLECULAR IMAGING

The presence of the BBB represents a challenge for molecular imaging of the brain. Indeed, to reach parenchymal targets, contrast agents should be able to cross the BBB. To date, there is no contrast agent able to cross the BBB in sufficiently large quantities to induce significant changes in MRI signal. Therefore molecular imaging of the brain is limited to endothelial proteins (such as VCAM-1, ICAM-1, E-selectin, and P-selectin), to diseases with compromised BBB (such as stroke, multiple sclerosis or severe traumatic brain injury) and to cells capable of crossing the BBB (such as monocytes). The purpose of this paragraph is to present the main issues related to each imaging targets that should be kept in mind when performing molecular MRI of brain–immune interactions.

Endothelial proteins are targets easily accessible to large contrast-carrying particles and have been accordingly successfully imaged using different contrast agents (from small GBCA to large iron oxide particles). The first issue related to imaging of endothelial targets is the requirement of a high affinity of the pharmacophore for its target because of the force exerted by the blood flow. These shear forces promote detachment of the contrast agent from the endothelial wall and thus limit the time window for imaging (von Zur Muhlen et al., 2008). Moreover, since MRI should be performed after complete washout of the contrast agent from the blood, a short half-life of the contrast agent is required to allow imaging before its detachment from the endothelium. Therefore, for efficient detection of endothelial targets, the contrast agent should combine a high affinity for its target and a short half-life. Importantly, to avoid false positive findings, the molecular weight of the contrast agent should be large enough to prevent passive extravasation across the BBB. Of note, once in the brain parenchyma, low molecular weight compounds are able to spread throughout the brain thanks to the glymphatic system (Gaberel et al., 2014).

Issues related to imaging of intraparenchymal targets in conditions of injured BBB are numerous. First, the molecular weight of the contrast agent should be low to maximize its passive extravasation through the BBB. The drawback is that this limits the quantity of contrast material carried by contrast agent molecules. In fact, only GBCA have been used for this purpose. Second, the specificity of this method is limited: even in the absence of the molecular target, the contrast agent accumulates in an unspecific manner in the brain regions with BBB leakage. Activatable contrast agents such as bis-5HT-DTPA(Gd) (a substrate for MPO) might partially overcome this limitation (Breckwoldt et al., 2008). Enzyme processing induces oligomerization and protein binding of the contrast agent leading to accumulation and increased T1 effect. Indeed, the T1 effect of paramagnetic contrast agents increases in parallel with the molecular weight due to limitation of motion of the Gadolinium atom. Therefore processed contrast agent molecules induce more important changes in signal intensity, allowing reduction of the injected dose and thus reducing unspecific signals. Nevertheless, interpretation of changes in signal intensity after contrast agent injection is difficult since it depends both on the importance of the BBB leakiness and on the concentration of the molecular target. Accordingly, post-contrast images would be similar in a condition of severe BBB impairment without MPO secretion and in a condition of mild BBB impairment with MPO secretion. This lack of specificity remains one of the main limitations of molecular MRI of parenchymal targets, even using activatable contrast agents.

**FIGURE 4:**
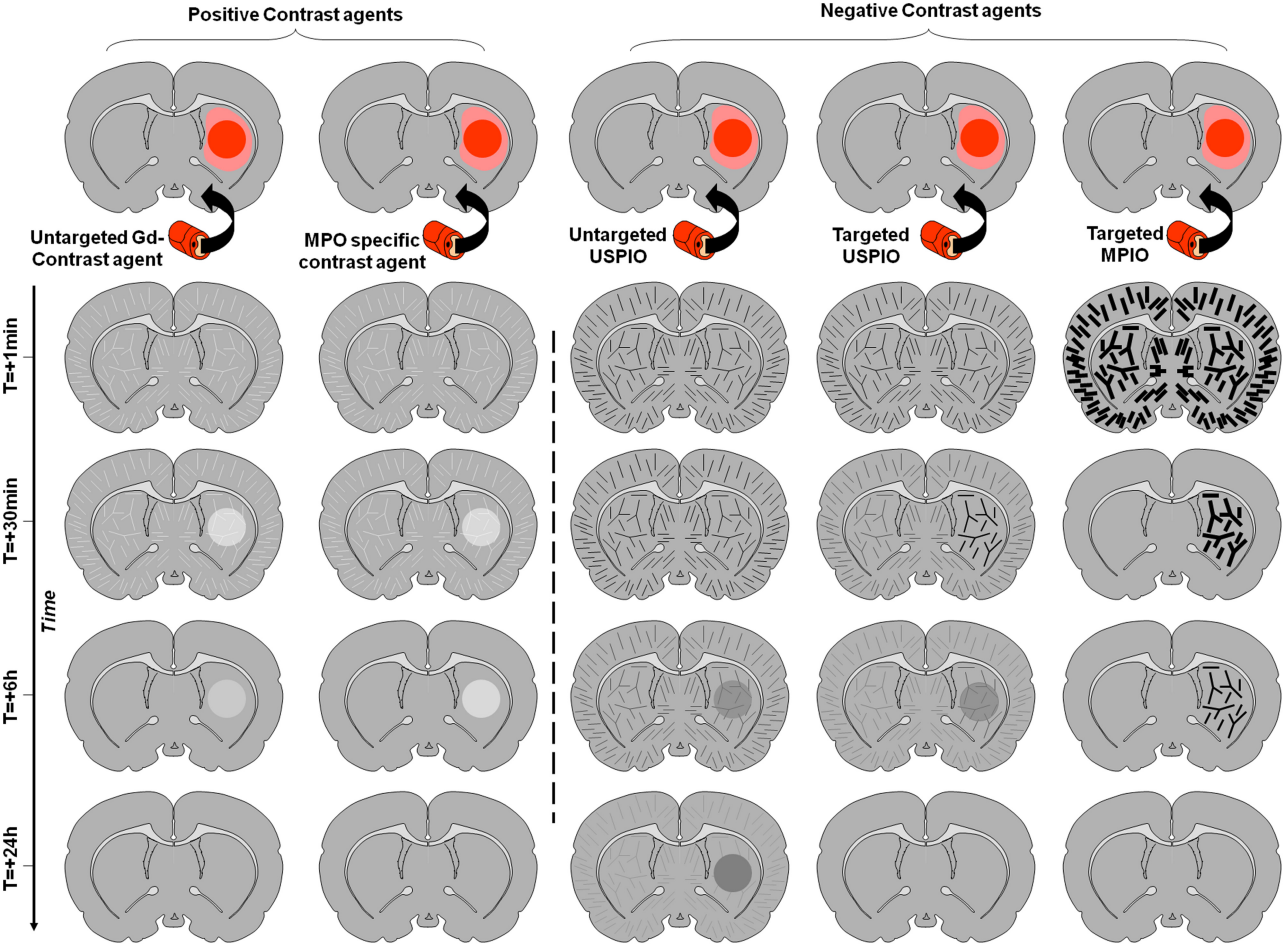
**Schematic representation of the effect of different contrast agents on brain MRI of a rodent with inflammation in the right striatum.** The inflammatory region with impaired BBB is represented in dark red, whereas the inflammatory region without BBB impairment is in light red. Time-dependent signal changes after contrast agent injection are represented vertically. In contrast to the signal changes induced by targeted MPIOs which are independent of the BBB status, the contrast enhancement induced by other contrast agents is not inflammation specific and strongly depends on the permeability of the BBB.

Imaging of leukocyte diapedesis is the third strategy developed for molecular MRI of the brain. Accumulation of leucocytes (especially blood-derived monocytes/macrophages) in the brain is a specific feature of neuroinflammation and then represents an attractive target for molecular imaging. However, this is a relatively delayed event after injury and requires a delay between injection of the contrast agent and imaging in order to allow its uptake and accumulation of leukocytes within the parenchyma. The prototypic contrast agents used to label leukocytes are unlabeled ultrasmall particles of iron oxide (USPIO; Corot et al., 2004). These small-sized iron-oxide-based contrast agents are internalized by circulating monocytes after intravenous injection. USPIOs-labeled monocytes are then detected by MRI once they have accumulated in the inflamed region of the brain. The delay between injection and imaging is usually long, about 24 h, limiting the interest of such contrast agents in the acute phases. Moreover, the relative amount of contrast agent eventually reaching the parenchyma is directly dependent on the number of monocytes entering the brain, thus limiting the sensitivity of the method. The other limitation is the possibility of false positive findings in case of BBB leakage, since USPIO readily crosses the BBB after stroke as well as in active plaques of multiple sclerosis for instance (Desestret et al., 2009). The different contrast appearances and the influence of the BBB status are illustrated on **Figure 4**.

In the next paragraphs, we will present the main studies reporting the use of molecular MRI for studying brain–immune interactions after acute cerebral injury (**Table 1**).

**Table 1:** List of all studies reporting *in vivo* molecular MRI of brain immune interactions in the last 15 years.

Target	Contrast carrying particle	Targeting moeity	Species	Experimental model	Reference
Macrophages	USPIO (Sinerem) diameter: 35 nm	Untargeted	Male Wistar rats	Ischemic Stroke: transient MCAo (60 min) by intraluminal filament	Farr et al. (2011)
Macrophages	USPIO (Sinerem) diameter: 35 nm	Untargeted	Male Lewis rats	EAE model: induction by 20mg of guinea pig myelin basic protein	Oude Engberink et al. (2010)
Macrophages	USPIO G 534-70 (ferumoxtran,Sinerem) diameter: 35 nm	Untargeted	Male Wistar rats	Ischemic Stroke: photo-sensitive dye rose induced a ischemic lesion in the right sensorimotor cortex	Saleh et al. (2004b)
Macrophages	USPIO AMI-227 (Sinerem) diameter: 40 nm	Untargeted	Male Fischer rats	Ischemic Stroke: transient MCAo (30 min) by intraluminal filament	Rausch et al. (2002)
Macrophages	Anionic iron oxide nanoparticles (AMNP)	Untargeted	Sprague-Dawley rats	Ischemic Stroke: transient MCAO (60 min) intraluminal filament	Riou et al. (2013)
Macrophages	USPIO AMI-227 (Sinerem) diameter: 40 nm	Untargeted	Female Lewis rats	EAE model: induction with xenogenic CNS proteins (guinea pig spinal cord).	Rausch et al. (2004)
Macrophages	VSOP (VSOP-R1 ) diameter: 7 nm	Untargeted	Female SJL/J mice	EAE model: induction 200μg of PLP (proteolipid protein)	Millward et al. (2013)
Macrophages	SPIO (Feridex) diameter: 60 μm	Untargeted	Male C57BL/6J mice	Traumatic Brain Injury model: induced by a controlled cortical injury.	Mills et al. (2012)
Macrophages	USPIO AMI-227 (Sinerem) diameter: 40 nm	Untargeted	Rat	Ischemic Stroke: cortical ischemic lesion (Rose Bengal)	Schroeter et al. (2004)
Macrophages	USPIO AMI-227 (Sinerem) diameter: 40 nm	Untargeted	Male Lewis rats	EAE model: induction with xenogenic CNS proteins (guinea pig spinal cord).	Rausch et al. (2003)
Macrophages	USPIO AMI-227 (Sinerem) diameter: 40 nm	Untargeted	Male Fischer rats	Ischemic Stroke: permanent MCAO by electrocoagulation	Rausch et al. (2001)
Macrophages	USPIO AMI-227 (Sinerem) diameter: 40 nm	Untargeted	Female Lewis rats	EAE model: induction with xenogenic CNS proteins (guinea pig spinal cord).	Dousset et al. (1999b)
Macrophages	SPIO (Resovist) diameter: 40 nm	Untargeted	Female rats	EAE model: induction with human MOG	Ladewig et al. (2009)
Macrophages	MPIO diameter: 0.9 μm	Untargeted	Male C57BL/6J mice	TBI model: induced by a controlled cortical injury.	Foley et al. (2009)
Macrophages	USPIO AMI-227 (Sinerem) diameter: 40nm	Untargeted	Male Swiss mice	Ischemic Stroke: permanent MCAO	Desestret et al. (2009)
Macrophages	USPIO AMI-227 (Sinerem) diameter: 40 nm	Untargeted	Female Lewis rats	EAE model: induction with myelin basic protein (MBP)	Baeten et al. (2008)
Macrophages	USPIO (Sinerem) diameter: 40 nm and SPIO (Endorem) diameter: 120 nm	Untargeted	Male Lewis rats	Ischemic Stroke: photo-sensitive dye rose induced a cortical ischemic lesion	Oude Engberink et al. (2008)
Macrophages	USPIO Ferumoxtran-10 (Sinerem) diameter: 35 nm	Untargeted	Male Swiss mice	Ischemic Stroke: permanent MCAO by electrocoagulation	Wiart et al. (2007)
Macrophages	USPIO AMI-227 (Sinerem) diameter: 40 nm	Untargeted	Female DA rats	EAE model: induction with xenogenic CNS proteins (guinea pig spinal cord).	Brochet et al. (2006)
Macrophages	USPIO AMI-227 (Sinerem) diameter: 40 nm	Untargeted	Female Lewis rats	EAE model: induction with xenogenic CNS proteins (guinea pig spinal cord).	Dousset et al. (1999a)
Macrophages	USPIO AMI-227 (Sinerem) diameter: 40 nm	Untargeted	Female Lewis rat	EAE model: induction with xenogenic CNS proteins (guinea pig spinal cord).	Berger et al. (2006)
Macrophages	USPIO 7228 diameter: 30 nm	Untargeted	Male Lewis rats	EAE model: induction with myelin basic protein (MBP)	Floris et al. (2004)
Macrophages	SPIO Feridex diameter: 40 nm	Untargeted	Male SHR/NCrl rats	Ischemic Stroke: transient MCAo (30 min) by intraluminal filament	Henning et al. (2009)
Macrophages	USPIO AMI-227 (Sinerem) diameter: 40 nm	Untargeted	Human	Stroke	Saleh et al. (2007)
Macrophages	USPIO SHU555C diameter: 25 nm	Untargeted	Human	Multiple sclerosis	Vellinga et al. (2008))
Macrophages	USPIO SHU555C diameter: 25 nm	Untargeted	Female DA rats	EAE model: induction with rat MOG	Chin et al. (2009)
Macrophages	USPIO SHU555C diameter: 25 nm	Untargeted	Human	Multiple sclerosis	Vellinga et al. (2009)
Macrophages	SPIO (Resovist) diameter: 40 nm	Untargeted	Male Wistar rats	Ischemic Stroke: cortical ischemic lesion (Rose Bengal)	Kleinschnitz et al. (2003)
Macrophages	SPIO (Resovist) diameter: 40 nm	Untargeted	Male rats	Ischemic Stroke: transient MCAo (60 min) by intraluminal filament	Kim et al. (2008)
Macrophages	USPIO AMI-227 (Sinerem) diameter: 40 nm	Untargeted	Human	Stroke	Nighoghossian et al. (2007)
Macrophages	SPIO (Resovist) diameter: 40 nm	Untargeted	Female DA rats	EAE model: induction with human MOG	Ladewig et al. (2009)
Macrophages	SPIO (Resovist) diameter: 40 nm	Untargeted	Male Sprague Dawley rats	Peripheral and central nerve injury: left sciatic and optic nerves were crushed	Bendszus and Stoll (2003)
Macrophages	USPIO AMI-227 (Sinerem) diameter: 40 nm	Untargeted	Human	Multiple sclerosis	Dousset et al. (2006)
Macrophages	USPIO (ferumoxtran-10) diameter 30 nm	Untargeted	Human	Multiple sclerosis	Manninger et al. (2005)
Macrophages	USPIO (Sinerem) diameter: 30 nm	Untargeted	Human	Severe internal carotid artery (ICA) stenosis	Trivedi et al. (2006)
Macrophages	USPIO AMI-227 (Sinerem) diameter: 40 nm	Untargeted	Human	Socioeconomic deprivation in elderly stroke/transient ischaemic attack and control medical patients	Cho et al. (2007)
Macrophages	USPIO AMI-227 (Sinerem) diameter: 40 nm	Untargeted	Male C57Bl/6J mice	Ischemic Stroke: Transient Middle Cerebral Artery Occlusion by clip (30 or 60 mins)	Denes et al. (2007)
Macrophages	monocrystalline iron oxide nanocolloid diameter: 4 nm	Untargeted	Male hypertensive rats	Ischemic Stroke: occlusion of the right middle cerebral artery by homologous blood clot	Dijkhuizen et al. (2002)
Macrophages	USPIO (Sinerem) diameter: 18 nm	Untargeted	Human	Stroke	Kooi et al. (2003)
Macrophages	USPIO	Untargeted	Sprague–Dawley rats	Ischemic Stroke: transient MCAO intraluminal filament	Yang et al. (2013)
Macrophages	SPIO (Resovist) diameter: 40 nm	Untargeted	Male pigs	hemorrhage model: sonication to open the BBB transcranially	Liu et al. (2011)
Mononuclear cells	VSOP C200 diameter: 62 nm	Spleen-derived MNCs	129/SV mice or C57/B6-GFP	Ischemic Stroke: transient MCAo (30 or 60 min) by intraluminal filament	Stroh et al. (2006)
T cells	SPIO (Ferumoxides) diameter: 120 nm	T-Cell isolated lymph node cells	Female SJL mice	EAE model: induction of PLP (proteolipid protein)	Anderson et al. (2004))
CD4+ T cells	USPIO (MACS) diameter: 30 nm	Antibodies against CD4+ T cells	Sprague–Dawley rats	ALS model: expressing mutated (G93A) human SOD-1	Machtoub et al. (2011)
CD4+ T cells	USPIO (MACS) diameter: 30 nm	Antibodies against CD4+	Female Wistar rats	Ischemic Stroke: global cerebral ischemia reversible by cardiac arrest	Sekeljic et al. (2012)
ICAM-1	MPIO (ProMag) diameter: 2 μm	Monoclonal antibody targeting ICAM-1 (YN1/1.7.4)	Female C57BL/6J mice	EAE model: induction with MOG	Blezer et al. (2014)
ICAM-1	Paramagnetic polymerized liposomes diameter: 200 nm	Monoclonal antibody targeting ICAM-1	SJL/J mice	EAE model: induction of PLP (proteolipid protein)	Sipkins et al. (2000)
ICAM-1	MPIO (ProMag) diameter: 1.05 μm	Monoclonal antibody targeting ICAM-1 (YN1/1.7.4)	Male C57Bl/6 mice	Ischemic Stroke: transient MCAO (30 min) by intraluminal filament	Deddens et al. (2013)
ICAM-1	SPIO diameter: 120 nm	Antibody targeting ICAM-1 (BD Biosciences)	Female Lewis rats	EAE model: induction with xenogenic CNS proteins (guinea pig spinal cord)	Schneider et al. (2009)
VCAM-1	MPIO (MyOne) diameter: 1.08 μm	Monoclonal antibody targeting VCAM-1 (clone A429)	Male C57Bl/6 mice	Ischemic Stroke: Permanent (electrocoagulation, FeCl3) and transient MCAO (Thrombin, Mechanical); Hemorrhage model: striatal injection of collagenase type VII and exogenous blood	Gauberti et al. (2013)
VCAM-1	MPIO (myOne) diameter: 1.08 μm	Monoclonal antibody targeting VCAM-1 (clone M/K2)	Male C57Bl/6 mice	Ischemic Stroke: transient MCAO (30 min) intraluminal filament	Hoyte et al. (2010)
VCAM-1	MPIO (myOne) diameter: 1.08 μm	Monoclonal antibody targeting VCAM-1 (clone M/K2)	Female SJL mice (8–12 wk old, 25 g; Harlan, Bicester, UK)	EAE model: induction of PLP (proteolipid protein)	Serres et al. (2011)
VCAM-1	USPIO (P03007) diameter: 26 nm	Monoclonal antibody targeting VCAM-1 (MCA 2297)	Male Swiss albino mice	Ischemic Stroke: transient MCAO (60 min) intraluminal filament	Fréchou et al. (2013)
VCAM-1	MPIO (myOne) diameter: 1.08 μm	Monoclonal antibody targeting VCAM-1 (clone M/K2)	Female adult Biozzi antibody high (ABH) mice	CR-EAE model: induction of mouse spinal cord homogenate	Mardiguian et al. (2013)
VCAM-1	MPIO (myOne) diameter: 1.08 μm	Monoclonal antibody targeting VCAM-1 (clone A429)	Male C57BL6/J mice	Alzheimer’s disease model: APP/PS1 mice Vascular dementia: Unilateral common carotid artery ligature EAE model: induction with MOG (rMOG 1-125)	Montagne et al. (2012)
P-selectin	SPIO (MNP-NH2) diameter: 50 nm	Sialyl Lewis X (sLeX)	Male C57/BL 6 mice	Ischemic Stroke: transient MCAO intraluminal filament	Jin et al. (2009)
Selectin (P-E)	GD-DTPA (Magnevist). Gd-DTPA-B(sLeX)	Sialyl Lewis X (sLeX)	Male C57 mice	Ischemic Stroke: transient MCAO (30 min) intraluminal filament	Barber et al. (2004)
E-selectin	USPIO	Heptapeptide for E-selectin targeting (IELLQAR)	Female Sprague–Dawley rats	TBI model: fluid percussion	Chapon et al. (2009)
PECAM-1	MPIO (ProMag) diameter: 1.05 μm	Monoclonal antibody targeting PECAM-1	Male C57Bl/6 mice	Ischemic Stroke: transient MCAO (30 min) intraluminal filament	Deddens et al. (2013)
Myeloperoxidase (MPO)	bis-5HT-DTPA(Gd)	bis-5HT-DTPA(Gd)	Female SJL mice	Venous stenosis model: ligation of the right and left jugular veins EAE model: induction of PLP (proteolipid protein)	Atkinson et al. (2012)
MPO	DTPA(Gd)	bis-5HT	Female SJL mice	EAE model: induction of PLP (proteolipid protein)	Forghani et al. (2012))
MPO	DTPA(Gd)	bis-5HT	Male New Zealand rabbits	Aneurysm model: induction by Elastase	DeLeo et al. (2009)
MPO	DTPA(Gd)	bis-5HT	Male C57/mice	Ischemic Stroke: transient MCAO (30 min) by intraluminal filament	Breckwoldt et al. (2008)
MPO	DTPA(Gd)	bis-5HT	Female SJL mice	EAE model: induction of PLP (proteolipid protein)	Chen et al. (2008)

## MYELOPEROXIDASE IMAGING

Myeloperoxidase is an abundant enzyme expressed by activated inflammatory cells of the myeloid lineage (Bradley et al., 1982), especially macrophages, monocytes, and neutrophils. MPO can induce endothelial dysfunction and increased inflammation due to upregulation of inducible nitric oxide synthase and carbamylation of lipoproteins. Indeed, MPO interacts with hydrogen peroxide to generate highly reactive species, such as OCl^-^, O_2_^-^, ONOO^-^ that can covalently modify lipids, causing further local tissue damage and perpetuating the inflammatory cascade (Klinke et al., 2011). MPO-generated free radicals induce apoptosis and nitro-tyrosination of proteins (Heinecke, 2002). Therefore, MPO is included in a complex cascade of inflammatory events involving different types of cells and molecules. Accordingly, molecular imaging of MPO could help detecting areas of brain inflammation (endogenous microglia and infiltrated macrophages/neutrophils) in several CNS disorders.

The idea to non-invasively “image” inflammation by targeting MPO expression/activity had first been investigated by Chen et al. (2004), in a key article in which the authors synthesized and tested a series of activatable paramagnetic MR contrast agents. After several developments, the same team selected a candidate, called bis-5HT-DTPA(Gd) (Querol et al., 2005, 2006).

Basically, a specific substrate of MPO (5-hydroxytryptamide) is associated with a Gadolinium-based contrastophore which is responsible for the paramagnetic effect. In the presence of MPO, the 5-hydroxytryptamide moiety of bis-5HT-DTPA(Gd) is oxidized and radicalized by hydrogen peroxide. The radicalized bis-5HT-DTPA(Gd) molecule can react with another radicalized bis-5HT-DTPA(Gd) molecule to form a polymer of up to five subunits leading to an amplification of the enzymatic reaction. In mice, the activated agent can also bind to proteins within inflammatory sites, trapping the agent, further increasing its molecular weight, and increasing the signal intensity on T1-weighted MRI (Chen et al., 2006). Converted products are locally retained, so that pharmacokinetics properties are improved: prolonged enhancement can be detected for up to 60 min at sites of increased MPO activity. The specificity of Bis-5HT-DTPA(Gd) was demonstrated using a substrate not specific for MPO (di-tyrosine), a pharmacological inhibitor of MPO (ABAH) or MPO knockout mice. In conclusion, bis-5HT-DTPA(Gd) appeared as a biocompatible agent with good sensitivity and specificity. Accordingly, this agent has been tested in several pathological contexts.

In experimental autoimmune encephalomyelitis (EAE) induced with synthetic proteolipid protein, a model of multiple sclerosis, MPO imaging has been tested for its ability to detect active plaques before clinical symptoms appearance in mice. The authors found that bis-5HT-DTPA allows detection of MPO, as a surrogate marker of the appearance and size of active plaques, and was correlated to clinical grading (Chen et al., 2008). In the same model of multiple sclerosis, another group used MPO imaging as an objective evidence of the therapeutic efficiency of an inhibitor (ABAH) of MPO. Bis-5HT-DTPA-based signals on imaging were smaller and matched with less demyelination and reduced severity of symptoms (Forghani et al., 2012). A study proposed that multiple sclerosis could be due to chronic cerebrospinal venous insufficiency (CCVI). However, bis-5HT-DTPA imaging performed in EAE mice subjected to ligature of the jugular vein to induce CCVI invalidated this hypothesis. Indeed, the authors did not observe any effect of CCVI on the correlation between MPO activity and neuroinflammation, demyelination, or clinical signs (Atkinson et al., 2012). Whether these negative results could be explained by the low sensitivity of this imaging method to detect subtle inflammatory changes remains, however, unclear.

Bis-5HT-DTPA has also been used in a model of transient focal stroke (transient mechanical vascular occlusion model during 30 min). In this study, MPO imaging correlated with infarct size, and the authors hypothesized that MPO imaging could be used to select and stratify patients for clinical trials of therapies targeting inflammation (Breckwoldt et al., 2008). In an experimental model of common carotid artery aneurysm induced by elastase, MPO imaging was shown to be valuable to identify unstable aneurysms, at risk of subsequent rupture (DeLeo et al., 2009).

Myeloperoxidase imaging has thus proven relevant to diagnose, predict, or evaluate therapeutic efficiencies in several models of CNS disorders. However, as already mentioned, it is widely recognized that these MRI techniques have limitations because contrast enhancement reflects breakdown of the BBB with leakage of paramagnetic chelates rather than active inflammation, and the two may not always correspond. Another potential limitation is that MPO imaging does not discriminate between MPO secreted from resident microglia, infiltrated macrophages or neutrophils.

Finally, the activated MPO sensor is cleared from the brain within 6 h after administration; the protein-bound, activated MPO agents are likely digested and released by proteases that are present at sites of inflammation (Querol et al., 2006).

Therefore, enzymatic imaging targeting MPO points to an interesting technology for non-invasive confirmation of active inflammatory lesions in brain disease. This could potentially not only improve disease diagnosis and treatment assessment in the clinical setting, but may also lead to a better evaluation of drug development and clinical trials of new therapies. This method is, however, impaired by its lack of specificity due to passive extravasation and accumulation of the unprocessed contrast agent, which contributes significantly to MRI signal changes.

## IMMUNE CELL TRACKING

Administration of exogenously labeled cells and subsequent tracking by non-invasive imaging has encountered numerous successes in molecular imaging of brain immune interactions. Unlike *in vivo* labeling of circulating cells using untargeted USPIO, this method has the advantage of a higher specificity since the contrast agent particles are clustered inside immune cells and are thus less susceptible to reach the brain in an unspecific manner (such as passive extravasation through a leaky BBB). Moreover, it allows direct imaging of brain–immune interactions as a whole, taking into account all the processes involved in the regulation of endothelial binding and blood to brain diapedesis of autologous cells. The basis of this method is to harvest autologous cells (most frequently leukocytes) and to label them with a high payload of contrast agent without impairing their ability to reach the CNS. Several labeling methods have been performed, based either on phagocytic uptake of contrast agent particles or on direct transfection of the cells with iron particles or Gadolinium using permeating agents.

One of the first studies investigating the use of systemically injected autologous labeled cells for molecular imaging demonstrated that rat bone marrow stromal cells were able to invade a photochemically induced brain lesion 7 days after intravenous injection (Jendelová et al., 2004). Later, Stroh et al. (2006) labeled spleen-derived mononuclear cells (MNCs) with very small superparamagnetic iron-oxide particles and transfused them into recipient mice subjected to transient ischemic stroke (filament model). Areas of signal hypointensities started to appear 24–48 h after intravenous injection of MNCs and corresponded to grafting of the cells in the ischemic lesion.

A similar method has been used by Anderson et al. (2004) in a mouse model of EAE by adoptive transfer of encephalitogenic T cells. They labeled endosomes from murine T-cells *ex vivo* using polylysine and ferumoxide and injected the labeled cells intraperitoneally in recipient mice. The authors were able to detect both *ex vivo* and *in vivo* the labeled cells in the lumbar spinal cord of EAE mice after the onset of clinical signs. Interestingly, the primary location of the encephalitogenic T-cells revealed by molecular MRI in this study was later confirmed by Arima et al. (2012), who demonstrated that autoreactive T cells access the CNS via the fifth lumbar spinal cord. Other immune-related cell-types can be labeled and subsequently imaged using the same labeling strategy, such as bone marrow-derived cells (Anderson et al., 2005).

Besides these proof-of-concept studies, molecular imaging of exogenously labeled cells allows to easily investigate the mechanisms driving the interactions between circulating cells and the brain endothelium. For instance, Gorelik et al. (2012) demonstrated in rats that iron oxide labeled glial-precursor cells can be targeted to the brain by the virtue of the interaction between circulating cell expressed VLA-4 and endothelial VCAM-1. Using MRI, they compared the efficiency of brain delivery of VLA-4 transfected and control glial precursor cells with or without VCAM-1 overexpression in the brain induced *in situ* by lipopolysaccharide injection. They found that brain delivery was dramatically enhanced when the injected cells and the brain expressed VLA-4 and VCAM-1, respectively. This kind of clever experiment can be performed using various types of circulating cells to improve our knowledge of brain–immune interactions.

The main limitation of this method is its reduced applicability for diagnostic purposes in humans. Although radiolabeling of autologous cells has been used successfully for imaging inflammation using SPECT and PET (Pulli and Chen, 2014), the limited sensitivity of MRI requires high payload of contrast agent per cell and a large amount of cells to be reinjected to achieve reliable imaging. Moreover, labeled cells might lose their iron oxide particles or die leading to false negative results. Another limitation is the effects of the labeling procedure including cell collection, purification, iron-oxide particles engulfment, and reinjection which can all influence the phenotype of the cells. To make this procedure faster and overcome some of its drawbacks, endogenous labeling of the monocyte/macrophage lineage using USPIOs has been developed.

## UNLABELED USPIO

Unlabeled USPIOs are virus-sized molecules with a very long blood clearance time (over 24 h). Iron oxide agents have greater contrast sensitivity compared to GBCA, generating signal voids on MRI, due to shortening of T2 and T2* relaxation times. A large range of sizes exist, from nano- to micron-sized iron oxide particles. USPIOs are among the most famous MR contrast agents, with a range in size from 10–50 nm, and should be differentiated from the larger SPIOs and MPIOs with diameters about 50–300 nm and 0.9–5 μm, respectively. Iron oxide particles are based on magnetite (Fe_3_O_4_), and are usually encased in polysaccharide, synthetic polymers, or monomer coatings (Thorek et al., 2006; Laurent et al., 2008). The utility of these particles as MRI contrast agents has been studied for more than two decades and the list of available agents is still expanding.

Ultrasmall superparamagnetic particles of iron oxides are readily internalized by phagocytic cells such as the Kupffer cell of the liver, circulating monocytes/macrophages and mononuclear T cells, as well as reactive astrocytes, microglia, and dendritic cells within the CNS. The USPIOs are cleared from the circulation primarily by the reticulo-endothelial system (Bourrinet et al., 2006); these properties (i.e., degree of cellular uptake and rate of clearance) are dependent on size, coating, and method of delivery.

The USPIOs ferumoxytol (Feraheme^®^, *AMAG Pharmaceuticals Inc*) was initially approved for iron-replacement therapy in patients with chronic renal failure. However, ferumoxytol has also been investigated in humans for various CNS imaging applications (Neuwelt et al., 2007), as well as ferumoxtran-10 (Manninger et al., 2005; Saleh et al., 2007) and SHU 555 C (Vellinga et al., 2008) to name a few. Ferumoxytol, in particular, is attractive as an MRI contrast agent because it is approved for use in humans and it is safe in patients with chronic kidney disease. It can be given as a bolus for first-pass perfusion imaging and at a later time point (e.g., 24 h) ferumoxytol accumulation is obvious in regions of BBB dysfunction that may be related to inflammation from any cause.

Several groups have shown that molecular imaging is an important new diagnostic tool for studying *in vivo* cellular and molecular biology across a wide range of disciplines, especially regarding inflammatory processes and associated immune cells, in both humans and animal models. Such applications may be relevant for earlier disease detection, more precise prognosis, personalized treatment strategies, monitoring of treatment efficiency and finally to improved our understanding of how cells behave and interact in their microenvironment *in vivo* (Thorek et al., 2006).

The most common strategy for cellular MRI of neuroinflammation involves tagging of circulating monocytes by systemic injection of iron oxide nanoparticles. In 2001, Rausch and coworkers first developed this approach in a stroke model in rats, where a single dose of USPIOs (Sinerem^®^, *AMAG Pharmaceuticals Inc*) was injected intravenously 5 h post-onset (Rausch et al., 2001). An accumulation of USPIOs was shown in the periphery of the lesion 24 h post-injection. At day 7, the USPIOs signal was still present and spread out within the lesion area, spatially correlating with areas displaying activated macrophages. Several groups have used a similar protocol to assess the spatiotemporal profile of monocyte infiltration in various stroke models in rodents (Kleinschnitz et al., 2003; Saleh et al., 2004b; Wiart et al., 2007; Kim et al., 2008). These studies raise an interesting technical point related to the USPIOs uptake by circulating white blood cells and, therefore, the USPIOs blood half-life. It has been shown that phagocytic cellular uptake of iron oxide increases with particle size (Daldrup-Link et al., 2003; Matuszewski et al., 2005). With a hydrodynamic diameter of 10 to 50 nm, USPIOs are less efficiently phagocytosed than SPIOs with sizes of 50–300 nm. The maximum intracellular iron oxide concentration of *in vitro*-labeled, isolated human macrophages is 50 pg iron/cell for the SPIOs ferucarbotran (Resovist^®^, *Bayer Schering Pharma AG*) whereas for USPIOs SHU 555 C (Supravist^®^, *Bayer Schering Pharma AG*), it is below 8 pg iron/cell (Metz et al., 2004). Besides the particle size of the iron oxide, phagocytic uptake is also dependent on nanoparticle surface properties (e.g., neutral versus charged). Numerous studies employed neutrally charged dextran-coated USPIOs (e.g., Sinerem^®^) with a long blood half-life compared to larger ferumoxides, such as dextran- (Endorem^®^in Europe, Feridex^®^in the USA) or anionic-carboxydextran- (Resovist^®^) coated SPIOs (Weissleder et al., 1989; Rausch et al., 2002; Briley-Saebo et al., 2006). Thus, the USPIOs extended blood half-life is assumed to promote the uptake by circulating cells. Furthermore, pharmacological strategies, using the protamine sulfate peptide, may be also used to enhance USPIOs uptake, as reported *in vitro* (Arbab et al., 2004) and *in vivo* in rats (Wu et al., 2007).

Although internalization of contrast agents in activated macrophages and its correlation with MRI contrast have been reported in different studies, there is still debate on the specificity of the observed MRI contrast. For instance, some studies suggested that USPIOs-induced contrast could be non-specific, i.e., due to a passive diffusion of iron particles through the BBB (Kleinschnitz et al., 2003; Saleh et al., 2004a; Denes et al., 2007; Nighoghossian et al., 2007; Saleh et al., 2007). The passive diffusion is patently increased because of a disrupted BBB in a context of stroke (Dijkhuizen et al., 2002). To distinguish between USPIOs passive diffusion in the brain parenchyma and inflammation, USPIOs-based MRI have been combined with Gadolinium-enhanced MRI to correlate USPIOs accumulation and BBB integrity, both in animal models and in humans (Kleinschnitz et al., 2003; Saleh et al., 2004a, 2007; Denes et al., 2007; Nighoghossian et al., 2007). As an alternative to the approach of labeling within the bloodstream, blood cells may also be extracted, labeled *in vitro* and then reinjected as presented above (exogenously labeled cells). This would avoid non-specific free iron particles leakage over the BBB and/or entrapment in the vasculature (Stroh et al., 2006; Oude Engberink et al., 2008). One of the latest unlabeled USPIO is P904 (Guerbet), for which phase I clinical trial has been initiated, demonstrating a significantly higher uptake by macrophages because of their pegylated coating compared to dextran-coated USPIOs (Yancy et al., 2005; Sigovan et al., 2009).

Despite the technical issues described above, this cellular MRI approach has given encouraging results to pursue further investigations. Non-targeted USPIOs may also be interesting in the field of atherosclerosis (including intracranial atherosclerosis), as suggested by experimental *in vivo* studies in hyperlipidemic rabbits (Ruehm et al., 2001; Briley-Saebo et al., 2008) and in humans (Kooi et al., 2003; Trivedi et al., 2006). These groups have shown non-targeted USPIOs accumulation in atherosclerotic plaques rich in macrophages, both in animals and humans. Additionally, USPIOs were shown to localize to atherosclerotic plaques-containing macrophages in apolipoprotein E knockout mice (Morris et al., 2008). Using the same transgenic model, a p38 MAP kinase inhibitor has been shown to decrease USPIOs uptake by atherosclerotic plaques, which correlated with a reduction in macrophage activity by histology.

## TARGETED USPIO

To counteract potential problems related to untargeted-USPIOs, targeted-nanoparticles of iron oxide have been developed to image neuroinflammation. VCAM-1 which promotes monocytes recruitment to the vascular wall and subsequent lesion development, is a promising marker for molecular imaging of cerebrovascular inflammation in several CNS disorders, since it is not constitutively expressed in normal vessels but is rapidly up-regulated on activated vascular endothelial cells (Davies et al., 1993; Cybulsky et al., 2001). On this basis, several generations of VCAM-1 targeted (using monoclonal antibodies or peptides generated by phage display) iron oxide nanoparticles conjugated with fluorescent molecules (Cy5.5) have been produced and applied in mouse models of acute inflammation and atherosclerosis (Kelly et al., 2005; Tsourkas et al., 2005; Nahrendorf et al., 2006). Molecular imaging of endothelial activation has been performed using USPIO labeled with a specifically designed targeting moiety consisting in a small peptide which triggers internalization of the USPIO by activated endothelial cells (Kelly et al., 2005). This amplification strategy allowed efficient intravital microscopic detection of the particles but this method lacked sensitivity for reliable *in vivo* imaging. Moreover, USPIOs conjugated to a VCAM-1 specific cyclic peptide (P03011 or R832, Guerbet) have recently been developed for *in vivo* detection of inflamed vessels in early and advanced atherosclerotic plaques by ultra-high field strength MRI (Burtea et al., 2012; Michalska et al., 2012), as well as in an ischemic stroke model in mice (Fréchou et al., 2013). Again, the sensitivity and specificity of VCAM-1 imaging using these USPIO-based strategies remained limited.

Among the different target of labeled USPIOs, E-selectin has been extensively studied in peripheral inflammation in mice (ear inflammation, muscle inflammation …; Reynolds et al., 2006). Selectins have also been investigated as target for MR molecular imaging of inflammation owing to their role in recruiting immune cells (i.e., leukocytes) to the vascular wall. Platelet-selectin (P-selectin), for example, is involved in early events of the inflammatory pathway, which make them an ideal target for early diagnosis of vascular inflammation. In addition, its baseline expression is near zero, which enables subtle changes to be detected in the vascular wall. E-selectin targeting is usually achieved using a Sialyl-Lewis X targeting moiety (USPIO-sLeX; Radermacher et al., 2009). But other USPIOs have been developed with peptidic (Chapon et al., 2009) or F(ab’) – based targeting moieties (Reynolds et al., 2006, Radiology). A study reported the use of E-selectin targeted USPIOs to detect endothelial activation in a mouse model of traumatic brain injury (Chapon et al., 2009). However, the sensitivity achieved using this agent is limited and does not allow reliable detection of brain E-selectin. A similar approach with P-selectin as a target has been developed and tested in an ischemic stroke model in mice (Jin et al., 2009), but presents the same limit as E-selectin.

While targeted USPIO-based molecular imaging remains an active field of investigation, targeted-USPIOs may have potential for toxicity, the sensitivity remains low even when amplification strategies are used and the specificity is impaired in condition of BBB leakage because of passive USPIOs extravasation. In addition, the long clearance time of USPIOs delays imaging until several hours after administration, which is not ideal for clinical use. For all these reasons, the feasibility of reliable *in vivo* molecular MRI using targeted USPIOs remains elusive.

## TARGETED MPIOs

Micro-sized particles of iron oxide have singular properties for endothelial cell-specific molecular imaging. First, their micron size range allows endovascular specificity unlike USPIOs agents which are susceptible to passive diffusion within the brain parenchyma, passive accumulation in atherosclerotic plaques or even non-specific macrophage uptake. Second, MPIOs convey a large payload of iron oxide (usually 0.1–1.6 pg Iron/MPIO particle), which is an order of magnitude larger than USPIOs contrast agents, resulting in strong hypointense contrast effects that may extend up to 50 times the physical diameter of the particle. This phenomenon, known as “blooming effect,” provides high sensitivity *in vivo* MRI detection, using only a small number of MPIO particles (Shapiro et al., 2005, 2006; Heyn et al., 2006). Third, MPIOs have very short blood half-life of 50–100 s (Ye et al., 2008; Yang et al., 2010), allowing imaging immediately after injection (Montagne et al., 2012; Gaberel et al., 2013; Gauberti et al., 2013). Finally, MPIOs are readily functionalized by covalent conjugation of ligands (monoclonal antibodies or their immunospecific fragments F(ab), single chain antibodies or peptides derived from phage display) to functional groups (amine, carboxyl acid or *p*-toluene sulphydryl (tosyl)) on the MPIOs surface.

Limitations of these agents for CNS imaging are related to their non-biodegradable coat and their potential iron toxicity. Indeed, MPIOs’ coated-sheath includes inert polymers giving them a non-biodegradable nature. Micro-sized particles may therefore accumulate in the reticulo-endothelial system (McAteer et al., 2007, 2008). To overcome this problem, more related to a clinical use, development of biodegradable MPIOs is currently in process, and some have already been developed (Nkansah et al., 2011). Although commercially available MPIOs did not show any side effects in cultured hepatocytes in terms of iron homeostasis and cell survival (Raschzok et al., 2011), these particles might have a potential long-term toxicity in humans due to the iron. Furthermore, the dose of iron used in most of experimental studies using MPIOs is around 4 mg iron/kg (McAteer et al., 2007), which is higher than the dose used in clinical practice to visualize tumors with USPIOs (2.6 mg iron/kg; Will et al., 2006). In addition, although their large size improves the specificity of MPIO-based molecular MRI, they are not able to enter the brain parenchyma and their targets are therefore limited to proteins expressed by the endothelium such as cell adhesion molecules.

Microsized particles of iron oxides have been applied for imaging of inflammation in many experimental studies, including various animal models of CNS disorders. Due to its properties mentioned earlier, VCAM-1 is an interesting biomarker in the field of molecular imaging and has been thoroughly studied over the last years. For instance, MPIOs targeting VCAM-1 has been studied in mouse models of acute cerebral inflammation (McAteer et al., 2007, 2012), chronic cerebral hypoperfusion (Montagne et al., 2012), atherosclerosis (McAteer et al., 2008), strokes (Hoyte et al., 2010; Gauberti et al., 2013), myocardial ischemia (Grieve et al., 2013; von Elverfeldt et al., 2014), Alzheimer’s disease (Montagne et al., 2012), multiple sclerosis (Serres et al., 2011; Montagne et al., 2012), and even in normal aging and systemic challenges related to risk factors of CNS disorders (e.g., peripheral inflammation, ethanol consumption, and hyperglycemia; Montagne et al., 2012).

**FIGURE 5:**
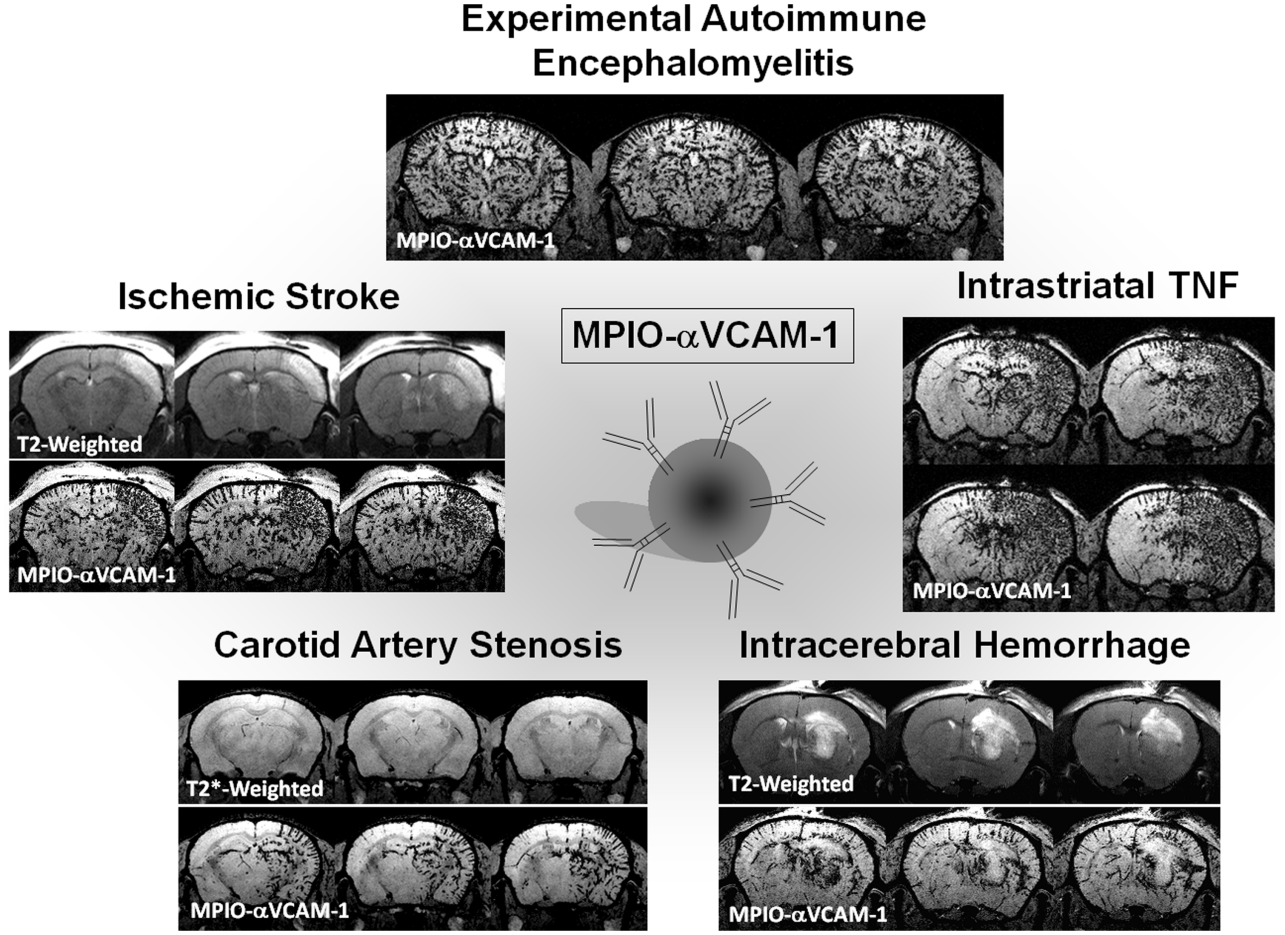
**Example of ultrasensitive molecular MRI of brain inflammation using optimized MPIO-αVCAM-1.** All the images presented were acquired 20 min after MPIO-αVCAM-1 injection as described by Montagne et al. (2012) and Gauberti et al. (2013). Experimental autoimmune encephalomyelitis was induced by Myelin oligodendrocyte glycoprotein (MOG) in C57BL6J mice. The T2*-weighted image presented is from a mouse with a clinical score of 3. Intrastriatal injection of TNF (1 μg) was performed 24 h before imaging. Intracerebral hemorrhage was induced by intrastriatal collagenase. Carotid artery stenosis was performed by ligation of the right common carotid artery. Ischemic stroke was induced by intra-arterial administration of thrombin.

Our group developed MPIOs conjugated to monoclonal VCAM-1 antibodies (MPIOs-αVCAM-1) and used them for non-invasive and high-sensitive *in vivo* detection of cerebrovascular inflammation in pre-clinical models in mice (Montagne et al., 2012). They provide higher sensitivity than previously reported methods and molecular contrast agents (McAteer et al., 2007), and are able to detect changes at a time that is otherwise undetectable using conventional MRI. Using an antibody anti-VCAM-1 carefully preselected by histology and a small dose of MPIOs (e.g., 1 mg iron/kg), we reported a dramatically higher sensitivity to assess cerebrovascular cell activation compared to previously published studies (**Figure 5**). In a model of acute inflammation in mice receiving a microinjection of pro-inflammatory cytokine tumor necrosis factor (TNF) into the right striatum, while the left striatum was not injected and served as internal control, we reported that *in vivo* T2*-weighted MRI reveals a potent hypointense contrast effect in the TNF injected hemisphere after MPIOs-αVCAM-1 injection 24 h post-onset. No contrast effects were seen in the non-injected hemisphere or in animals injected with control MPIOs-IgG. Similarly, although pre-contrast MRI images failed to reveal the ongoing pathology, contrast-enhanced MRI using MPIOs-αVCAM-1 revealed hypoperfusion-triggered CNS injury in vascular dementia, unmasked amyloid-induced cerebrovascular activation in Alzheimer’s disease and allowed monitoring of disease activity during EAE (Montagne et al., 2012).

Other vascular biomarkers (mostly cell adhesion molecules) have been studied over the last few years as potential targets for MR molecular imaging of inflammation. ICAM-1, unlike VCAM-1, is constitutively expressed in endothelial cells. ICAM-1 is nevertheless up-regulated during an inflammatory response. Deddens et al. (2013) studied ICAM-1 up-regulation after stroke in mice using a similar approach based on MPIOs coupled to anti-ICAM-1 antibodies. Using the same custom contrast agent, this group also showed ICAM-1 overexpression during EAE, providing an early tracer of disease activity (Blezer et al., 2014). McAteer et al. (2012) constructed dual antibody-conjugated MPIOs, targeting both VCAM-1 and P-selectin (50:50 ratio). They did not show further enhance contrast effects compared to single VCAM-1 antibody-conjugated MPIOs in a model of acute inflammation in mice (McAteer et al., 2012). Endothelial-Selectin (E-selectin) has also been investigated *in vitro* using similar dual antibody-conjugated MPIOs, targeting both VCAM-1 and E-selectin (Jefferson et al., 2011).

Microsized particles of iron oxides are well-tolerated in mice, with no short-term ill effects. Clearance experiments showed that MPIOs are sequestered by the liver and spleen 24 h after injection, with no evidence of adverse effects such as tissue infarction, inflammation, or hemorrhage. The MPIOs used so far in experimental studies are non-biodegradable, due to their inert coat. However, the basic iron contrast mechanism is potentially transferable to humans with suitable adaptation of the surface coat.

Their size similar to circulating platelets, their short blood half-life and the exceptionally potent T2* MRI contrast effects of targeted MPIOs, together with advancement of biocompatible MPIOs, provide translational imaging opportunities for improved diagnosis and treatment of endovascular inflammation in various CNS disorders. However, extensive toxicological profiling and synthesis of sterile, clinical-grade targeted MPIOs according to good manufacturing practice will be required before clinical translation is feasible.

## LAST AND FUTURE DEVELOPMENTS

To date, molecular MRI studies consisted primarily on proof-of-concept studies reporting the feasibility to image a particular target. Very few studies used molecular MRI as a tool to investigate brain–immune interactions. Reasons for this are numerous including the lack of reliability of the imaging procedures with the already mentioned false negative (mainly due to a lack of sensitivity of most molecular MRI procedures) and false positive results (due to passive extravasation through an altered BBB, endogenous contrast induced by microhemorrhages or iron accumulation, inadequate post-processing analyses…). In particular, most of the MRI contrast agents are described in unique studies and no confirmatory results in other models or by other groups are presented. Altogether, these drawbacks limited the confidence of the molecular imaging community in molecular MRI and finally raised doubts about its feasibility. Of note, the recent use of MPIO-based contrast agents dramatically improved the reliability of molecular MRI and the latest studies were able to provide new insights into brain immune interactions.

**FIGURE 6:**
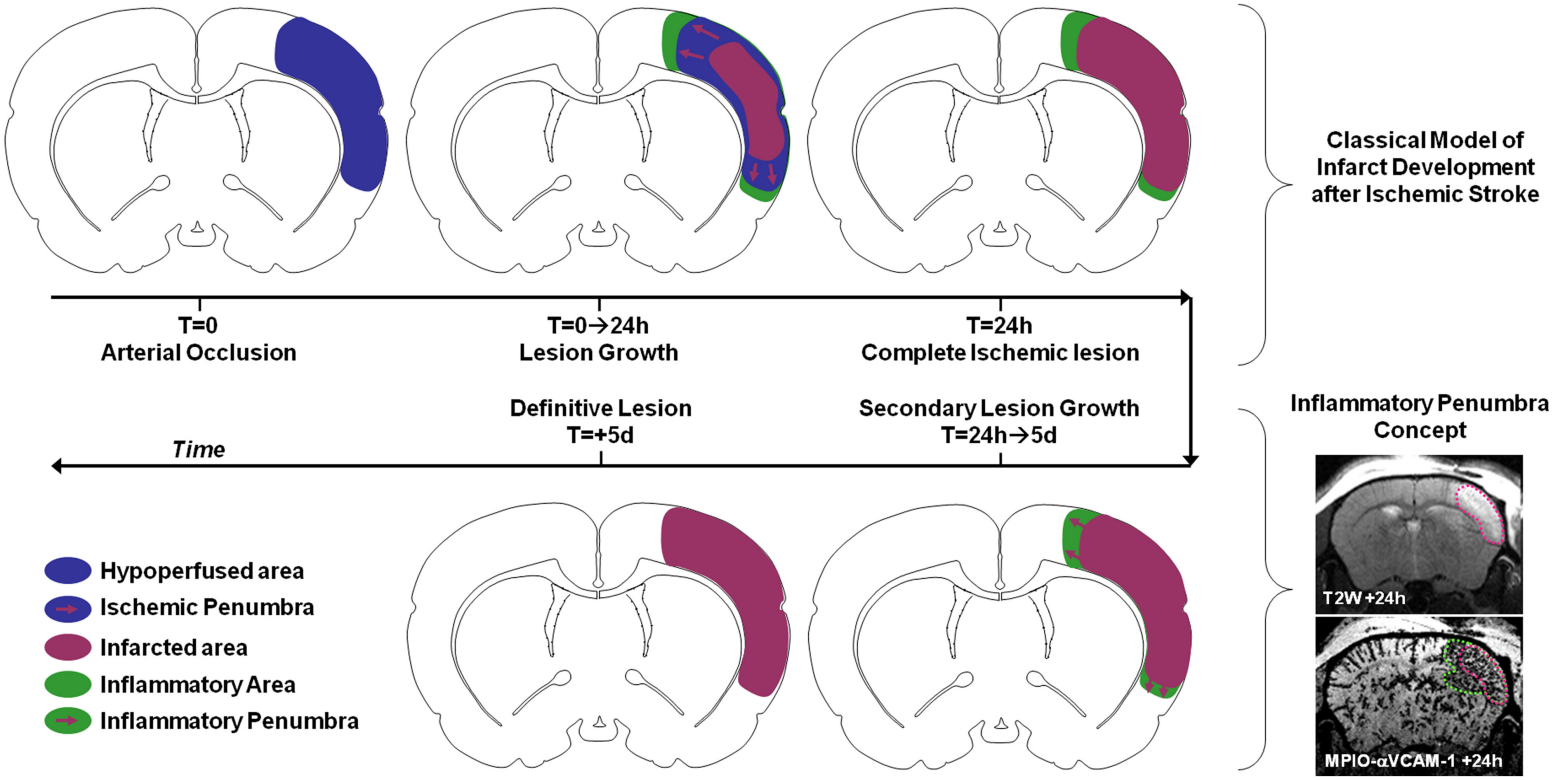
**Schematic representation of the inflammatory penumbra concept after ischemic stroke as revealed by MPIO-αVCAM-1 enhanced MRI.** VCAM-1 overexpression is sustained and particularly significant in the peri-infarct area after ischemic stroke. From 24 h to 5 days post-stroke onset this inflammatory penumbra is recruited by the ischemic core. Interestingly, anti-inflammatory treatments block this secondary infarct expansion. In this context, the presence of an inflammatory penumbra 24 h after stroke onset revealed by molecular MRI of VCAM-1 may be used as a selection criteria for anti-inflammatory treatment in stroke patients.

For instance, numerous studies investigated the impact and timing of leukocyte diapedesis after ischemic stroke. However, the spatiotemporal modulation of the expression of adhesion molecules by the brain endothelium after ischemic onset was unknown. Using MPIOs-αVCAM-1 in mice, we demonstrated that stroke induced overexpression of VCAM-1 (involved in monocyte and T-Cell diapedesis) not only in the ischemic lesion, but also in intact brain regions (Gauberti et al., 2013). In particular, VCAM-1 overexpression was very strong in the close periphery of the lesion. Using longitudinal imaging, we were able to demonstrate that this overexpression was sustained for 5 days after permanent ischemia (by electrocoagulation or permanent thrombosis of the middle cerebral artery) but much quicker after transient ischemia (Gauberti et al., 2014; Le Behot et al., 2014). Interestingly, when VCAM-1 overexpression is sustained, the corresponding inflammatory region is recruited by the ischemic core in a delayed fashion. Since this secondary lesion growth can be blocked by anti-inflammatory treatments (such as celecoxib or high-dose statins) and by analogy with the ischemic penumbra, we proposed the concept of inflammatory penumbra to describe this peri-lesional area overexpressing VCAM-1 (**Figure 6**). This pathophysiological concept is supported by other studies demonstrating the critical role of VCAM-1 dependent T-cells diapedesis in delayed lesion growth after ischemic stroke (Liesz et al., 2011). In our study, the ability to simultaneously and longitudinally observe VCAM-1 expression and ischemic lesion development offered by molecular MRI was critical to determine the fate of the inflammatory penumbra. In a clinical setting, such imaging strategy may help to discriminate patients who will experience malignant stroke characterized by an exacerbated inflammation (Vivien et al., 2011). Experimental studies on the impact of peripheral inflammation on brain–immune interactions also benefited from molecular MRI. It is for instance possible to reveal the impact of sepsis, acute hyperglycemia, acute ethanol intoxication, or aging on brain endothelium expression of VCAM-1 using MPIO-based molecular MRI in mice (Montagne et al., 2012). The exceptional sensitivity of the method and its ease of use offer new avenues of investigations in the field of neuroinflammation.

Given the huge improvement in sensitivity and specificity of molecular imaging of inflammation offered by MPIO-based molecular MRI, efforts are ongoing to adapt this method for clinical imaging. Indeed, currently used MPIOs are not biodegradable, especially because of their poly-urethane coating. Therefore, the development of biodegradable and biocompatible MPIOs is mandatory for further development of this imaging technology. For instance, the production of multimeric magnetite particles forming large MPIO-like particles which are biodegradable has been described (Nkansah et al., 2011). Another active subject is the impact of MPIO binding on endothelium physiology. Indeed, several studies demonstrated outside-in signaling after binding of leukocytes on ICAM-1 expressing endothelial cells. ICAM-1 clustering due to leukocyte binding triggers intra-cellular calcium release and subsequent activation of RhoA and the formation of actin stress filaments (Jefferson et al., 2013). Concerning VCAM-1, a recent *in vitro* study demonstrated that MPIOs labeled with VCAM-1 targeting antibodies do not promote endothelial inflammation, in contrast to monocytes (Jefferson et al., 2013). However, this potential side effect of targeted MPIOs should be tested for all endothelial targets before clinical investigations. The risk of immunization of the receiver of targeted MPIOs preparation must also be studied and potentially counteracted using non-immunogenic targeting moieties.

Besides toxicity issues, translation of MPIO-based molecular MRI from bench to bedside will require the demonstration of the feasibility to detect MPIOs using magnet at clinical field strength and using standard coils. Indeed, the sensitivity of MPIO detection depends both on the field strength (higher field strength would produce larger susceptibility effect of the MPIOs) and on the spatial resolution of the MRI acquisition. This last parameter would be crucial to optimize for clinical translation since the resolution achieved in preclinical study is below 80 μm (isotropic) and allows single MPIO imaging whereas the highest resolution of 3D T2*-weighted images on clinical magnet is around 500 μm. This lower resolution, because of partial volume effects, will lower the sensitivity of the MRI acquisition to detect MPIOs. To overcome this issue, longer acquisition times to increase the signal to noise ratio and strengthen the T2*-weighting will be necessary and could limit the clinical availability of this method. Interestingly, a whole brain 3D T2*-weighted image with an isotropic resolution of 500 μm can be acquired using a 7 Tesla clinical magnet in 6 min with a high signal to noise ratio using echo planar imaging (Zwanenburg et al., 2011). Such imaging method with a clinically compatible acquisition time would be particularly well suited for MPIO-based molecular MRI and argue toward the feasibility of sensitive and timely molecular MRI in humans. Studies in large animals such as non-human primates are awaited to demonstrate the feasibility of MPIO-based imaging in humans.

## CONCLUSION

While several years ago the feasibility of rapid and reliable molecular imaging of brain–immune interactions using MRI was elusive, the mini-revolution represented by the development of MPIO-based contrast agent has changed this view. Although molecular MRI using targeted MPIOs still remains restricted to preclinical imaging, studies aiming at closing the translational gap are ongoing. Once available, such contrast agents will undoubtedly help to improve our knowledge on the brain–immune interactions taking place in the human brain after acute neurological injury. Besides, they will represent potent tools to select patient for anti-inflammatory treatment and subsequently monitor the therapeutic response. Nevertheless, molecular MRI using targeted MPIOs is limited to imaging of endothelial targets and does not allow long term investigation of the fate of leukocyte once in the brain parenchyma. To this aim, exogenous labeling of autologous cells and subsequent cell tracking using MRI remains the method of choice. These two imaging strategies (i.e., targeted MPIOs and MRI cell tracking) appear today as the most promising technologies to non-invasively study immune cells trafficking in both preclinical and clinical studies. These tools will help to determine whether molecular MRI of brain–immune interactions can improve the management of patients presenting acute brain injury.

## Conflict of Interest Statement

The authors declare that the research was conducted in the absence of any commercial or financial relationships that could be construed as a potential conflict of interest.
